# Physiologically Difficult Airways in Emergency Medicine: A Narrative Review of Recognition, Resuscitation, and Management Strategies

**DOI:** 10.7759/cureus.103512

**Published:** 2026-02-12

**Authors:** Chetla Rakesh, Anagani Hrushikesh, Nandhini Shree, C.V.K.K Chaitanya, Vusirikayala Naga Sireesha, Naveen Mohan

**Affiliations:** 1 Emergency Medicine, Amrita Institute of Medical Sciences, Kochi, IND; 2 Department of Anesthesia, Centurion University of Technology and Management, Vizianagaram, IND; 3 Public Health, Amrita Institute of Medical Sciences, Kochi, IND

**Keywords:** critical illness intubation, emergency airway management, peri-intubation complications, physiologically difficult airway, pre-intubation resuscitation

## Abstract

The focus of managing critically ill patients' airways has traditionally centered around anatomically complicated issues, however an increasing amount of literature indicates that peri-intubation complications are likely to be related to the patient's physiologic state at the time of intubation. Physiologic instability may lead to severe hypoxemia, cardiovascular collapse, or cardiac arrest even when the anatomy of the airway is uncomplicated. Shock states, severe metabolic acidosis, right heart failure, or an insufficient oxygen reserve may contribute to these unstable conditions but they often remain undetected. The lack of inclusion of physiological instability as part of many modern predictive models, guidelines, and algorithms also contributes to the lack of a standard framework or standardized thresholds for using physiological data (oxygenation indices, hemodynamic profiles, acid-base status, bedside cardiac assessment) to determine optimal timing or techniques of airway management. The current paper reviews the available literature to identify the best methods for identifying and managing patients who have a physiologically difficult airway. These include performing an integrated assessment of the patient's physiology, optimizing both gas exchange and perfusion before inducing anesthesia, choosing induction strategies that will result in the least harm to the cardiovascular system, using multiple modalities of prediction instruments to assess the likelihood of developing physiological instability during airway management, utilizing point-of-care ultrasound, and employing awake or modified rapid sequence intubation techniques appropriately. A "physiology first" approach provides a model to decrease the incidence of hypoxemia and circulatory collapse during the process of airway management. To further develop and advance the field of emergency and critical care medicine, it is recommended to standardize the pre-intubation physiologic screen, incorporate proven physiologic markers into existing airway algorithms, and support the development of future research studies aimed at establishing standardized thresholds, improving existing prediction models, and providing guidance on pre-intubation optimization of critically ill and emergently ill patients in emergency and critical care.

## Introduction and background

Airway management is one of the essential elements of emergency medicine, critical care, and anesthesiology and is a high-stakes intervention capable of initiating severe adverse events in critically ill patients [1-3]. Historically, the construct of a challenging airway has been defined mainly by ways of anatomy that hinder adequate visualization of the glottis and successful tracheal tube insertion [4].

Although the airway devices and techniques, along with anatomy-based predictive algorithms, have advanced, critically ill patients are still facing high rates of perioperative complications associated with the use of the endotracheal tube. This is due to the fact that tube-placement-related adverse events have been reported in 20%-45% of instances, which makes it clear that direct glottic visualization and technical success are inadequate predictors of procedural safety [2,3].

On top of the anatomic hurdles, there is mounting evidence that major physiological derangements can significantly influence airway-related outcomes. Some clinical investigations have found right ventricular dysfunction and peri-intubation hypotension to be pathophysiological factors that may contribute to the cardiovascular collapse during airway management, even when the airway anatomy does not appear complex [5]. These findings highlight the limitations of relying solely on anatomical assessment and emphasize the importance of physiological evaluation in critically ill patients.

Traditionally, the use of anatomical factors has determined the airways assessment, but clinicians have used systematic predictive instruments to determine technical difficulty. Mnemonics like LEMON (Look externally; Evaluate; Mallampati; Obstruction or obesity; Neck mobility) can help to notice the characteristics of the restricted mouth opening, short thyromental distance, or limited neck movement, which can make airway management difficult [6,7].

In response to the limitations of anatomy-based models, Mosier et al. proposed the notion of a physiologically challenging airway in 2015 [1]. This system outlines patients with pronounced physiological lability in whom manipulation of the airways and the commencement of positive-pressure ventilation contribute significantly to the risk of cardiovascular collapse, even when the patients are thought to have easy intubation. Identification of this construct has contributed to the development of airway risk knowledge in addition to the conventional anatomical definitions.

Further studies have developed assessment tools that incorporate physiological variables into airway evaluation. As an example, Difficult Airway Physiological Score (DAPS) provides an organized dimension to combine physiological parameters in case of airway interventions [8], and reviews of point-of-care ultrasound expose its position as a procedure to assess anatomical and physiological airways [9]. The emphasis on maximizing pre-oxygenation and preventing peri-intubation desaturation has further highlighted the importance of physiological preparation in high-risk patients, including the use of continuous apneic oxygenation during the apneic phase of intubation to prolong safe apnea time and mitigate hypoxemia [10].

Combined with the above developments, it is clear that safe airway management in the critically ill involves more than knowledge of anatomical technique but an active positioning of the physiological condition of the patient. Having a complete appreciation of the two whole anatomical and physiological regions will help the clinician to foresee the complications to expect and adjust airway strategy to them. Accordingly, this review outlines the recognition of the physiologically difficult airway, strategies for pre-intubation optimization, airway management approaches, and emerging future directions relevant to emergency and critical care practice.

## Review

Methods

The current research was conducted as narrative synthesis as opposed to systematic review. To narrow the literature search, PubMed and Google Scholar were used as the main sources of information to locate the articles in the English language with high clinical relevance regarding airway management in adults with critical illnesses and published since January 2000 to March 2025. All reference lists of key articles and pertinent specialty society guidelines were checked manually to identify high-impact articles that might not have been accessed through database searching only. There was no risk-of-bias appraisal of risks in accordance with the narrative design or quantitative synthesis. 

Complex search strategies were developed using combinations of relevant keywords and Medical Subject Headings (MeSH), including “physiologically difficult airway,” “pre-intubation optimization,” “peri-intubation complications,” “emergency intubation,” and “hemodynamic management.” Representative search strategies included (“physiologically difficult airway” OR “physiologic airway risk”) AND (“emergency intubation” OR “critical care airway”), as well as (“peri-intubation hypotension” OR “peri-intubation hypoxemia”) AND (“airway management” OR “endotracheal intubation”).

Inclusion criteria included peer-reviewed original research papers, narrative reviews, as well as professional guidelines focused on physiologically challenging airways under emergency or critical care settings. Such exclusion criteria as non-English publications, case reports, conference abstracts, animal studies and studies that consider only anatomical airway difficulty but not physiological factors were used. Qualitative data extraction was conducted with the focus on the essential clinical themes which include predictors of physiological instability, pre-intubation optimization strategies, airway strategies, and peri-intubation outcomes. Results were presented in a narrative form through the synthesis of physiological principles embedded with observational studies results, clinical trials and guideline-based recommendations. 

Two independent reviewers filtered the records found during the search. Screening consisted of title and abstract screening, then full-text screening of potential eligible articles. The end product of evidence was a blend of original research articles, review of articles, and professional guidelines considered to be important in the management of difficult airways which were physiologically challenging to work with in the critical care units.

Recognition of the physiologically difficult airway

A clinically challenging airway can be recognized by an astute clinician paying attention to parameters suggesting when deterioration is more likely to occur during or after sedation. While anatomical difficulties may generally be "visually assessed" and assessed at the bedside, physiological disturbances often require a broader understanding of the patient’s general condition [1]. Early detection of physiological instability is essential because these derangements directly increase the risk of peri-intubation hypoxemia, hypotension, and cardiovascular collapse, even when laryngoscopy appears anatomically straightforward. The recognition framework can be best organized into key physiological derangements and validated predictive tools. Table 1 summarizes key physiological derangements [3-5], and Table 2 summarizes the predictive tools [4,6-9].

**Table 1:** Major physiological derangements associated with peri-intubation instability. SpO2- Peripheral Oxygen Saturation, V/Q - Ventilation-Perfusion, SBP – Systolic Blood Pressure, HR – Heart Rate, CO2 – Carbon dioxide, RV - Right Ventricular, PVR – Peripheral Vascular Resistance

Physiological Derangement	Clinical Indicators / Predictive Tools	Description / Significance	References
Hypoxemia	SpO2 < 92% or inability to oxygenate with SpO2 < 92%. Severe hypoxemia (SpO2 < 80%).	Critically ill patients desaturate rapidly due to pre-existing cardiopulmonary pathology, anaemia, low cardiac output, V/Q mismatch, and hypermetabolic states.	[3]
Hypotension	SBP < 90 mmHg or hypotension resistant to fluids requiring vasopressor therapy post-intubation. Shock Index (HR/SBP) > 0.8 or > 0.9.	Pre-intubation hypotension (SBP < 90 mmHg) is associated with a 12-15% incidence of peri-intubation cardiac arrest. Elevated shock index is an early warning sign of shock progression.	[4]
Severe Metabolic Acidosis	pH < 7.3.	Apneic interval can lead to a rapid and profound drop in pH due to CO2 accumulation, causing cardiovascular collapse, arrhythmias, and decreased myocardial contractility.	[4]
RV Failure	Identified by pre-intubation echocardiography.	RV is highly sensitive to changes in preload and afterload; intubation can catastrophically worsen its function. Induction agents decrease preload, apnea increases PVR, positive pressure ventilation decreases RV preload.	[5]

**Table 2:** Predictive tools for physiologically difficult airway assessment SpO2 - Peripheral Oxygen Saturation, GCS – Glasgow Coma Scale, ICU – Intensive Care Unit, LV – Left Ventricle, RV – Right Ventricle, DAPS - Difficult Airway Physiological Score, POCUS - Point-of-Care Ultrasound

Criteria	Parameters	Description	Reference
CRASH	Consumption (increased oxygen consumption), Right Ventricular Failure, Acidosis (severe metabolic acidosis), Saturation (severe hypoxemia), Hypotension.	Systematic tool for emergency physicians to consider and address critical physiological factors before intubation.	[4]
HEAVEN Criteria	Hypoxemia (SpO2 ≤ 93% at initial laryngoscopy), Extremes of size, Anatomical challenge, Vomit, blood, or fluid in the airway, Exsanguination/Anemia, Neck mobility issues.	Incorporates both anatomical and physiological elements to predict difficult airways in emergency settings. High negative predictive value (97%) for overall intubation difficulty.	[6]
MACOCHA Score	Mallampati score III or IV (5 points), Obstructive sleep apnoea (2 points), Reduced cervical spine mobility (1 point), Limited mouth opening <3 cm (1 point), Coma (GCS ≤8) (1 point), Severe hypoxemia (SpO2 <85% on room air) (1 point), Non-anaesthesiologist performing intubation (1 point).	Predicts difficult intubation in ICU patients; score ≥6 indicates higher likelihood. Sensitivity 75%, specificity 82%.	[7]
DAPS	12 pre-intubation clinical and laboratory parameters (e.g., age, gender, vitals, pH <7.3, shock index >0.9, anticipated patient decline).	Predicts serious outcomes (cardiac arrest, post-intubation hypotension, oxygen desaturation). Accuracy 77%, sensitivity 74%, specificity 83.3%.	[8]
POCUS	Assesses anterior tissue thickness (> 2-2.5 cm), hyomental distance (< 5.29 cm), tongue thickness (< 6.1 cm) for anatomical difficulty. Identifies LV/RV dysfunction, valvular abnormalities, pericardial effusion, fluid responsiveness for hemodynamic optimization.	Essential for predicting difficult airways and guiding management; provides real-time dynamic physiological parameters.	[9]

Clinicians should first assess the four physiological domains (Table 1). If any domain is abnormal, use prediction tools (Table 2) and POCUS when available to identify hemodynamic instability or right ventricular compromise. Scores like DAPS quantify physiological risk and can be used to trigger pre-intubation optimization - fluids, vasopressors, and targeted oxygenation. Emerging approaches, including machine-learning-based risk stratification models and advanced point-of-care imaging, may further enhance early identification of patients at high risk for peri-intubation physiological collapse.

Resuscitation strategies for the physiologically difficult airway

Breathy intubation should only be attempted in patients with a physiologically difficult airway once they have been pre-oxygenated for a period and their physiological parameters optimized. The intention is to stabilize critical derangements because intubation may worsen these derangements in order to create a safer environment for the procedure to take place. “Resuscitation Before Intubation” stresses the importance of airway attempts only following resuscitative efforts directed toward hemodynamic and oxygenation support. Pre-intubation optimization acts directly to mitigate peri-intubation hypoxemia, hypotension, and cardiovascular collapse. It should be done systematically before the administration of either sedative or paralytic agents.

The key components of pre-intubation resuscitation include optimization of oxygenation, hemodynamic stabilization, acid-base management, and physiological adaptation in the case of right ventricular failure. These interventions must be performed before laryngoscopy and not after the development of a complication.

Preoxygenation and Apnoeic Oxygenation

Maximizing oxygen stores, and subsequently the time to desaturation, is essential to all intubations, especially of the critically ill, and begins with enough preoxygenation. Normal healthy adults can safely undergo apnoea for eight to 10 minutes after optimal preoxygenation to 99% SpO2, compared to the one minute of safe apnoea time when breathing room air [10]. This provides an important backup to maintain the airway for longer and increases the chances of success on the first attempt as well as decreases the incidence of complications [11,12].

Hemodynamic Support

Hypotension was strongly associated with peri-intubation cardiac arrest and increased in-hospital mortality [11]. Intubate aggressively, but only after the hemodynamics are optimized [12]. This is physiologically evident, as induction agents, muscle relaxants, and positive pressure ventilation may acutely worsen hypotension through a vasodilatory effect, reduced venous return, and decreased cardiac output, respectively.

Management of Severe Metabolic Acidosis

Intubation of patients with more severe metabolic acidosis, such as those with a pH of less than 7.1, is very difficult and risky. They generally compensate by hyperventilating to "blow off" the CO2 and preserve the pH; intubation itself may create apnoeic intervals, which can significantly lower the pH as CO2 increases even in a brief interval, resulting in acidosis, arrhythmias, and decreased cardiac contractility [13].

Management of Right Ventricular Failure

Intubating a patient with right ventricular failure, for example due to a massive pulmonary embolism, poses a tremendous physiological challenge. The right ventricle (RV) is very sensitive to preload and afterload and intubation can dramatically compromise RV function.

Specific mechanisms by which intubation can harm a failing RV include vasodilation by induction agents which reduces preload (upon which the failing RV relies to maintain cardiac output), and apnoea which can result in hypoxemia, hypercarbia, and acidosis thereby increasing pulmonary vascular resistance (PVR) and RV afterload. PVR is volume-dependent and increased at both excessively low and excessively high lung volumes. Also, the switch to positive pressure ventilation results in additional reduction of venous return and RV preload [14]. Ongoing technological advances in oxygen delivery systems, hemodynamic monitoring, and ultrasound-guided resuscitation continue to refine physiologically informed airway preparation and warrant further investigation

Management strategies for the physiologically difficult airway

Once physiological instability has been identified and understood, airway management should be planned to minimize additional insult during induction and tracheal intubation. In patients who have a physiologically difficult airway, the choice of intubation technique and associated adjuncts is not strictly a technical decision but rather is integrally tied to the underlying derangements, including hypoxemia, hemodynamic instability, metabolic acidosis, and reduced physiological reserve. The aim of airway strategy selection is to maximize first-pass success (successful intubation on the initial attempt) while minimizing apnea duration, excessive blunting of sympathetic response, and repeated manipulation of the airway [15-20]. Table 3 lists the commonly employed intubation techniques and airway adjuncts, outlining their major indications, the physiological insult they prevent, and the principal limitations relevant to the critically ill.

**Table 3:** Intubation techniques and adjuncts for the physiologically difficult airway RSI – Rapid Sequence Intubation, SpO₂ – Peripheral Oxygen Saturation, RV – Right Ventricle, SGA – Supraglottic Airway Device.

Technique / Adjunct	Primary indication in physiologically difficult airway	Physiological risk addressed	Key advantages	Important limitations / cautions	References
Rapid sequence intubation (RSI)	Urgent need for airway control with risk of aspiration	Reduces apnea time and aspiration while maintaining rapid access to the airway	Familiar technique, quick airway control, widely available	Induction and paralysis can precipitate hypotension, loss of compensatory ventilation, and cardiovascular collapse in unstable patients.	[15]
Awake tracheal intubation	Severe hypoxemia, metabolic acidosis, or hemodynamic instability coupled with intolerance to apnea.	Preserves spontaneous ventilation and maintains airway tone	Avoids apnea and large hemodynamic fluctuations; allows for continued oxygenation	Need for patient cooperation, operator expertise, and additional time. Poorly tolerated in agitated patients.	[16]
Video laryngoscopy	Anticipated difficult visualization or a goal to maximize first-pass success	Decreases in prolonged laryngoscopy and numbers of repeated attempts	Better glottic visualization, and higher first-pass success rates in various clinical settings.	Failure to mitigate physiological instability and continued reliance on equipment and screen visibility	[17]
Bougie-assisted intubation	Poor glottic view or narrowly opening larynx.	Decreased number of repeated attempts and duration of airway manipulations	High first-pass success if used early; simple adjunct	Misplacement into esophagus and operator skill dependence.	[18]
Supraglottic airway device	Failed intubation or rescue oxygenation	Rapid restoration of oxygenation and ventilation	Fast deployment; useful bridge to definitive airway	Does not protect against aspiration; limited ventilation in severe lung pathology	[19]
Suction-Assisted Laryngoscopy Airway Decontamination (SALAD) Method	Massively contaminated airway due to active vomiting, hematemesis, regurgitation, or copious secretions during emergency intubation in unstable patients	Prevention of rapid hypoxemia, aspiration, and prolonged laryngoscopy caused by obscured glottic visualization in critically ill patients with limited physiological reserve	Allows continuous, aggressive suctioning during laryngoscopy; improves visualization of the glottis; reduces repeated intubation attempts and airway manipulation time; facilitates first-pass success in contaminated airways	Does not correct underlying physiological instability; requires operator training and coordination; effectiveness reduced in extremely high-volume bleeding; should be integrated with preoxygenation and hemodynamic optimization	[20]
Fibreoptic laryngoscopy	Anticipated difficult airway with severe hypoxemia, metabolic acidosis, or hemodynamic instability where preservation of spontaneous ventilation is critical; commonly used during awake tracheal intubation	Avoidance of apnoea-induced hypoxemia, hypercapnia, and cardiovascular collapse by maintaining spontaneous ventilation and airway reflexes	Permits controlled, awake intubation; minimizes hemodynamic fluctuations; useful in limited mouth opening, cervical spine instability, airway distortion, angioedema, or maxillofacial trauma; nasal or oral approach possible	Operator-dependent and time-intensive; limited utility in profuse bleeding or secretions; requires patient cooperation; not suitable for rapidly deteriorating or peri-arrest patients	[16]

A key concept is that no intubation strategy uniformly mitigates all physiologic risks; an inappropriate strategy can lead to the deterioration of hemodynamic stability even with technical success of the procedure. Strategies that preserve spontaneous ventilation, minimize apneic duration, or reduce the number of laryngoscopy attempts may be better for some patients, while there is the potential for rapid pharmacologic induction of cardiovascular decompensation in others. Accordingly, airway management should be personalized according to physiological assessment, operator’s expertise, and urgency of the clinical situation with the recognition of adjuncts augmenting and not replacing comprehensive pre‑intubation optimization and attentive post‑intubation monitoring. As airway management increasingly shifts toward a physiology-first paradigm, future innovations are expected to integrate real-time physiological data, advanced visualization, and decision-support systems to individualize airway strategies in critically ill patients.

Failed airway management and cricothyrotomy

Even with meticulous planning and skilled execution, airway management may fail. A failed airway is a life-threatening situation where tracheal intubation cannot be established despite proper attempts at using alternative techniques or where further attempts are abandoned in the face of increasing danger. The most critical manifestation is the cannot intubate, cannot oxygenate (CICO) scenario, which requires immediate emergency front-of-neck access (eFONA) to avoid hypoxic brain injury and death.

Because of the limited oxygen reserve, poor cardiovascular compensation, and intolerance of repeated manipulations in patients with a physiologically difficult airway, failure evolves more quickly. Early identification of failure and strict adherence to a planned rescuing protocol are consequently essential. The guidelines promulgated by the Difficult Airway Society and the American Society of Anaesthesiologists urge systematic and stepwise methodology with the aim of minimising the number of repetitive attempts, focusing on getting patients oxygenated and prescribing an expeditious transition to eFONA where clinically warranted [21]. Table 4 outlines key steps of the algorithm for failed airway management according to current emergency and critical care practice [21,22].

**Table 4:** Algorithmic approach to failed airway management and emergency front-of-neck access

Algorithmic Step	Description	Reference
Attempts	No more than 3 + 1 attempts at direct or video laryngoscopy should be performed, with the final attempt reserved for an expert operator. Repetitive attempts without modification are discouraged because they increase the risk of airway trauma and adverse outcomes.	[21]
Resuscitating	If intubation attempts are unsuccessful, immediate attention should shift to oxygenation using bag-mask ventilation or placement of a supraglottic airway device as a rescue strategy (Plan B).	[22]
“Stop and Think” strategy	When adequate oxygenation has been achieved using a rescue device, clinicians should pause to reassess the situation and consider waking the patient or proceeding with a more definitive airway plan rather than continuing repeated intubation attempts.	[21]
Emergency front-of-neck access (eFONA)	Emergency front-of-neck access, most commonly performed as an open scalpel cricothyrotomy, represents Plan D and is indicated in a cannot-intubate, cannot-oxygenate (CICO) scenario when both mask ventilation or supraglottic airway devices have failed. Needle cricothyroidotomy is less preferred due to lower success rates and a higher incidence of complications.	[21]

In those patients with physiological instability, strict adherence to airway management protocols is of special significance, considering that hypoxia or hypotension may cause rapid progression to cardiac arrest. To control secondary injury, an immediate progression to techniques of rescue oxygenation and avoiding repeated unmodified attempts against secondary insult are critical issues. Anticipation and early preparation for emergency front of neck access in cases where this is indicated (high risk) is recommended as the ability to perform this intervention in a timely fashion, and not as a delayed rescue, is the main determinant of survival in the cannot intubate cannot oxygenate scenario.

Post-intubation management

The outcomes associated with a physiologically difficult airway comprise more than the successful attainment of tracheal intubation; improper management in the post intubation period may lead to further physiological deterioration. Accordingly ventilatory settings should be individualised to the underlying derangement. In those patients who have marked metabolic acidosis, immediate post-intubation ventilation should be near the pre-intubation compensatory hyperventilation to prevent sudden increases in arterial carbon dioxide tension and associated worsening of acidosis. This often requires respiratory rates and volume breaths that are higher than those achievable using traditional/standard lung protective algorithms [1]. Conversely, patients who have right-ventricular dysfunction need ventilatory strategies that will allow minimization of mean airway pressure while eliminating hypoxemia, atelectasis, and hypercapnia, each of which increases pulmonary vascular resistance and increases right-ventricular afterload [23].

It follows that even with pre‑intubation optimization, hemodynamic instability remains common. Continuous hemodynamic monitoring is essential, and ongoing vasopressor or inotropic support should be maintained or escalated as necessary to prevent post‑intubation hypotension and cardiovascular collapse [24].

Special considerations in specific patient populations

While the basic principles guiding the management of the physiologically difficult airway are widely applicable, specific patient populations harbour unique anatomical, physiological, and situational challenges that increase the potential for deterioration around the time of intubation. Factors relating to trauma instability, reduced respiratory reserve, altered anatomy of the airway, and competing maternal-foetal priorities may necessitate special airway approaches beyond general methods. Appreciation of these population-specific factors is crucial for the modification of airway planning, limiting secondary injury, and improving outcomes in high-risk scenarios.

Trauma Patients

Trauma patients often have multiple physiological derangements (hypovolemia, haemorrhage, shock, acidosis etc.) and anatomical challenges (maxillofacial injury, airway distortion, cervical-spine injury, swelling etc.) that create a physiologically difficult airway. The task of securing an airway amid chaos and at the very least of time allowed for an optimization increase the chance of complications. In-line stabilization is necessary for patients with cervical instability. There has been concern that pre-hospital intubation, especially in the context of trauma and traumatic brain injury, is associated with poor prognosis, and that bag-valve-mask ventilation may be more beneficial than endotracheal intubation in some situations - in particular given the pernicious risks of hyperventilation and exacerbation of neurologic injury [23,25].

Obese Patients

The probability of both anatomically and physiologically difficult airways is substantially increased in obesity. Redundant fat of the upper airway, hypopharynx, and neck decreases pharyngeal space, compromises mask seal, and interferes with laryngoscopy visualization of the glottis, while tongue volume enlargement and soft-tissue redundancy further encroach on airway patency. Functionally, obese patients have decreased functional residual capacity, reduced chest wall compliance, higher oxygen consumption, and are prone to atelectasis, which causes them to desaturate rapidly after induction of anaesthesia. Collectively, these changes reduce the margin for apnoea, increasing the likelihood of peri-intubation hypoxemia and cardiovascular instability. Thus, preoperative preparation, optimized preoxygenation, and strategies aimed at minimizing apnea duration and repeated airway manipulation must be carefully integrated into the airway management of obese patients with physiological compromise. In this context, the provision of continuous oxygenation during the apneic phase of intubation, particularly using high-flow nasal oxygen, plays a critical role in prolonging safe apnea time and reducing the risk of rapid desaturation in this high-risk population [10,21,26].

Paediatric Patients

Airway management in the paediatric patient is complicated by several factors related to anatomical differences and limited physiological reserve. These factors include relatively large tongues, large adenoids and tonsils, and a larynx positioned more anteriorly and superiorly (approximately at the C3 vertebra in infants versus the C5 vertebra in adults). In particular, the challenges are more striking among neonates and infants, in whom the disproportionately large head, short slender neck, and relatively large tongue all act in concert to increase further the risk of difficult airway situations, as evidenced in current paediatric airway guidelines [27]. All these factors make obstruction of the airway and difficulty with visualization more likely. The paediatric airway is funnel-shaped, with the cricoid cartilage being the narrowest portion rather than the vocal cords. Children have limited apnoeic tolerance due to high metabolic rates, lower functional residual capacity, and increased susceptibility to respiratory muscle fatigue. Mask ventilation can easily lead to gastric insufflation, elevation of the diaphragm, and further decreases in lung volume, and a shorter trachea increases the risk of mainstem intubation or accidental extubation. All these factors, combined with epiglottitis, croup, or foreign body aspiration, greatly increase the potential for rapid deterioration during manipulation of the airway. Paediatric-specific data regarding the physiologically difficult airway are limited. Nevertheless, set principles stress the need for thorough planning, apnoeic intervals reduction and experienced people. Pediatric difficult airway guidelines promulgated by the Difficult Airway Society in collaboration with other international anaesthesia societies emphasize strategies of structured management of unexpected difficult airways [28,29].

Obstetric Patients

Pregnant patients pose unique challenges for managing the airways due to a combination of physiologic adaptations and anatomic changes associated with the physiologic urgency conferred in conjunction with a state of physiologic adaptation. Pregnancy is linked with reduced functional residual capacity, increased oxygen consumption and reduced tolerance to apnoea; hence, rapid oxygen desaturation occurs after induction of anaesthesia. The mucosa of the airways become more vascular and oedematous during gestation and thus instrumentation of the airway becomes more arduous and bloodier. The tone of the lower oesophageal sphincter is decreased and gastric emptying is delayed, hence further enhancing the risk of aspiration during airway manipulation. Gestational weight gain and breast hypertrophy may make it difficult to optimally position it and provide laryngoscopic access. Although progress in training, equipment, and standardised protocols has controlled the problem of obstetric airway complications, failure rates remain higher than in non-pregnant patients. Current guidelines recommend careful pre-intubation airway assessment, careful hospital risk reduction for aspiration, and accurate communication and record keeping after unsuccessful intubation to ensure the welfare of both mother and fetus [30].

Recent advancements and future directions

The future of airway management could be shaped by emerging technologies, but the importance of these technologies should be estimated in the framework of the existing physiological theory and the existing clinical evidence. Present developments are largely focused on creating better real-time physiological risk stratification, procedural planning, and decision support systems, and hence the necessity to carry out additional studies to understand how these tools can significantly reduce the occurrence of peri-intubation hypoxemia, hemodynamic collapse, and cardiac arrest in critically ill patients.

AI-driven predictive analytics are a very promising area of airway management. Machine learning models that combine patient characteristics, physiological parameters, and imaging information are expected to lead to better early identification of patients at higher risk for airway-related complications and to clinical decision-making. Innovations in imaging and visualization - impacting virtual endoscopy, based on three-dimensional computed tomography reconstructions - may be used as adjuncts to pre-procedure airway assessment by providing detailed anatomical models extending the standard bedside evaluation. Robotic and image-guided endoscopic systems are also being developed to improve the accuracy and stability of intubation, though their use in the emergency and critical care settings will have many constraints based on availability, cost, and need for further validation. Technological innovation has also concentrated on incremental development of existing airway devices. Contemporary endotracheal tubes have enhanced cuff designs that reduce aspiration and tracheal injury, whereas newer supraglottic airway devices boast improved sealing characteristics, with some having integrated gastric drainage that may widen their application as either rescue or bridging airways for unstable patients

Despite ongoing advances, significant knowledge gaps persist in the management of the physiologically difficult airway. Comparative effectiveness research continues to seek the best preoxygenation strategy for patients with severe hypoxemia, including investigation into the relative benefits of non-invasive positive-pressure ventilation compared to high-flow nasal oxygen. The role of prophylactic or early vasopressor therapy aimed at preventing peri-intubation hypotension in patients in shock is incompletely defined and merits further study. Awake tracheal intubation is increasingly recommended for select high-risk cohorts; however, well-designed comparative data to assess the use of awake techniques compared to Rapid Sequence Intubation (RSI) are lacking, particularly with respect to comparing the risk of aspiration and patient-centered outcomes. Prospective studies and updated clinical recommendations should focus on the use of standardized physiologic assessment, well-defined intervention thresholds, and outcome-oriented comparisons of airway strategies in a concerted effort to reduce peri-intubation morbidity and mortality.

## Conclusions

The identification of physiologically challenging airways has led to the shift in the approach of management of emergent airway toward a physiology-first paradigm, whereby systemic instability is given equal importance as anatomic obstacles. Hypoxemia, hypotension, severe metabolic acidosis, and right ventricular failure reliably predict peri-intubation complications and should be improved if possible before intubation. Effective management requires meticulous preparation, including advanced preoxygenation calibrated to the degree of respiratory compromise, the use of continuous apneic oxygenation during the intubation process, delivered via low-flow nasal cannula or high-flow nasal oxygen, to prolong safe apnea time and reduce peri-intubation hypoxemia, although the optimal modality remains an area of ongoing investigation, hemodynamic optimization guided by point of care ultrasound and meticulous post-intubation surveillance in order to preclude subsequent deterioration, primarily in the context of the presence of acidosis and right sided cardiac dysfunction. The use of structured instruments along with ultrasound such as HEAVEN, MACOCHA, and DAPS helps enhance the early identification of risks and ensures timely implementation of resuscitative measures. Clinicians have been recommended to think carefully before working on unstable airways with the understanding that correction of physiologic derangements prior to induction is essential in ensuring improved patient safety and reducing morbidity during and after intubation.
